# Quantification of Mg^2+^, Ca^2+^ and H^+^ transport by the gastrointestinal tract of the goldfish, *Carassius auratus*, using the Scanning Ion-selective Electrode Technique (SIET)

**DOI:** 10.1371/journal.pone.0207782

**Published:** 2018-12-04

**Authors:** Vladimir Kodzhahinchev, Andrew Biancolin, Carol Bucking

**Affiliations:** Department of Biology, York University, Toronto, Ontario, CA; Universitat Politècnica de València, SPAIN

## Abstract

An *in vitro* gut-sac technique and the scanning ion-selective electrode technique (SIET) were used to characterize Mg^2+^, Ca^2+^, and H^+^ transport at both the mucosal and serosal surfaces of non-everted and everted gastrointestinal tissues obtained from *Carassius auratus*. As part of the study, two magnesium ionophores were compared (II vs. VI). Unfed animals displayed uniform transport of all ions along the intestine. Feeding resulted in elevated Mg^2+^ and Ca^2+^ transport when the gut lumen contained chyme however, under symmetrical conditions this increased transport rate was absent. Furthermore, zonation of divalent cation transport was present for both Ca^2+^ and Mg^2+^ under non-symmetrical conditions while the zonation remained for Ca^2+^ alone under symmetrical conditions. High dietary Mg^2+^ decreased absorption and induced secretion of Mg^2+^ in the posterior intestine. Uptake kinetics in the esophagus suggest large diffusive and/or convective components based on a linear relationship between Mg^2+^ transport and concentration and lack of inhibition by ouabain, an inhibitor of Na^+^-K^+^-ATPase. In contrast, kinetics in the rectum were suggestive of a low affinity, saturable carrier-mediated pathway. A decrease in Mg^2+^ and Ca^2+^ transport was observed in the posterior intestine (both at the mucosal and serosal surfaces) in response to ouabain. This impact was greatest for Ca^2+^ transport and when applied to the mucosal fluid and measured in everted preparations. In contrast a putative Mg^2+^ transport inhibitor, cobalt(III)hexamine-chloride, did not affect Mg^2+^ transport. This is the first study to use SIET approaches to study ion transport in the gut of teleost fish. This is also the first study to provide characterization of Mg^2+^ transport in the gut of *C*. *auratus*. Due to the limited selectivity of Magnesium ionophore II, subsequent studies of tissues bathed in physiological saline should be made using Magnesium Ionophore VI.

## Introduction

Freshwater teleosts inhabit hypoosmotic environments and face persistent ion loss, relying on continuous ion uptake to preserve homeostasis. Indeed, Mg^2+^ and Ca^2+^ absorption is critical for freshwater fish development, including skeletal formation and growth (e.g. [1, 2]). Active ion absorption by the gills, along with renal ion reabsorption from urine, are both the principal foci for previous transport studies. More recently, studies have highlighted dietary sources and the gastrointestinal tract (GIT) as a vital additional route for both Mg^2+^ and Ca^2+^ uptake (e.g. [3, 4, 5, 6]). In fact, 70–90% of Mg^2+^ requirements in freshwater fish is believed to be supplied by their diet [7], via the GIT [8, 9, 10]. These studies have treated the GIT as a homogenous organ. However, regional differences along the GIT in terms of morphology, enzyme activity, and transporter expression (e.g. [6, 11, 12]), illustrate the need to study these functional zones independently.

Teleost GIT transport studies employ traditional techniques namely; the gut sac (e.g. [12, 13, 14, 15]), Ussing chambers (e.g. [16, 17, 18]), and isolated brush border membrane vesicles (BBMVs; e.g. [19, 20, 21]). While these techniques have provided invaluable insight into GIT transport, each have critical limitations. The gut sac technique uses isolated segments of the GIT filled with known solutions, incubated in saline for several hours. The entire serosal and/or mucosal bath(s) are then measured for net ion movement. Gut sacs allow simultaneous studies of large sections of the GIT without disturbing the mucous layer of the tissue, have fast preparation times, and the required training and instrumentation is minimal. However, the technique does not provide fine-scale resolution of transport within a sac and requires larger animals (i.e. adult life stages and larger species) thereby limiting the model organisms and/or life stages that can be studied. In contrast, Ussing chambers can be used to study more localized transport rates depending on the aperture size, however, once again a reliance on larger and/or adult animals is required to obtain sufficient tissue for successful preparations. As well, Ussing Chambers produce more reliable, repeatable results when employing tissues with high transepithelial electrical resistance, limiting their use with more fragile and leaky tissues (i.e. the GIT) at least in mammals [22, 23, 24]. Finally, while isolation of BBMVs reveals either apical or basolateral transport, generating only partial knowledge of transport mechanisms without interaction between the two cell membranes (e.g. [20 vs 21]). A common limitation of all techniques is their reliance on radioisotopes to quantify the transport of a specific ion, restricting these techniques to the study of ions with a suitable radioisotope. Unfortunately, Mg^2+^ radioisotopes have short half-lives (11.9 sec for Mg^23^ and 9.8 min for Mg^27^) or are cost-prohibitive (Mg^28^; [25]). Thus, existing literature has focused on ions that can be measured with relative ease, creating a paucity of information on Mg^2+^ transport. Regardless of the specific approach, use of radioisotopes additionally demands careful handling, training, and costly disposal, as well as specific permits, due to the pronounced potential health and environmental risks.

The Scanning Ion-selective Electrode Technique (SIET) is a powerful tool for studying ion transport (explained in great detail in [26]). In brief, the technique relies on positioning a selective electrode (a glass capillary pulled into a fine tip containing an ion-selective ionophore) at distances from a desired point of measurement and comparing the measured voltage relative to a stationary reference electrode (an agar bridge kept away from the measurement point). The measured voltage gradient can then be converted into a concentration gradient, allowing determination of the ion transport’s magnitude and directionality. There are a number of ionophores, specific to numerous individual ions such as Mg^2+^ and Ca^2+^. Indeed, SIET has been extensively used for characterizing NH_4_^+^, Na^+^, Cl^-^, K^+^, and H^+^ transport across tissues in several insect species, including the anal papillae in the common mosquito (*Aedes aegypti*) and the midge (*Chironomous riparius*; e.g. [27, 28]). These studies also revealed zonation of transport along an organ structure on a microscopic scale, and pharmacologically characterized transport through the application of specific inhibitors (e.g. methazolamide on H^+^ and Cl^-^; [29]). Interestingly, SIET has successfully shown NH_4_^+^, H^+^, and Na^+^ transport across different areas of the skin in intact larval *D*. *rerio* [30, 31, 32]. We propose that SIET provides a useful method to study GIT transport as it: safely, rapidly, and accurately measures ion movement on a microscale in three dimensions, is well suited for studying transport and zonation along small organs increasing the number of model species that can be studied, and enables researchers to explore the relatively unexplored area of Mg^2+^ transport.

Here we used SIET in conjunction with gut sac preparations to study Mg^2+^, Ca^2+^, and H^+^ transport, as well as the impact of feeding, dietary treatment, and zonation of transport, along the GIT of *Carassius auratus*. We also used pharmacological agents and kinetics of Mg^2+^ transport to characterize transport in several areas of the GIT. The aim of the present study was to measure and compare the Mg^2+^, Ca^2+^, and H^+^ transport observed along the serosal and mucosal surfaces of the GIT from *C*. *auratu*s in fed and starved individuals using SIET. We hypothesized active, carrier-mediated transport kinetics in the intestine [2, 20], increases in transport rates in fed individuals [6], and inhibition of active transport using established inhibitors [20, 21, 33]. The results indicate that SIET offers a novel technique for studying ion transport in the teleost intestine at a microscale.

## Materials and methods

All reagents were purchased from Fisher Scientific (Fisher Scientific Co, Fair Lawn, New Jersey, USA) unless otherwise stated. All measurements were observed at room temperature (20°C). Experiments were conducted according to approved animal use protocols at York University (AUP 2015–12) in accordance with Canadian Council on Animal Care guidelines.

### Animal care

*C*. *auratus* (3 g– 12 g; Big Al’s; Toronto ON, CA) were housed in 50 L opaque tanks supplied with continuous aeration and flow-through dechlorinated City of Toronto water. Water temperature (20°C) was maintained with submersible heaters. The animals were exposed to a 12h:12h light:dark cycle. During acclimation to laboratory conditions, animals were fed to satiation daily with commercial fish feed (Wardley’s Goldfish Floating Pellets; Hartz, USA).

### Diets and sampling

Following laboratory acclimation, animals were fed daily to satiation with re-formed food pellets. Commercial pellets (Wardley’s Goldfish Floating Pellets) were crushed into a powder and reverse osmosis water (60% vol/weight) was added to make a paste. The paste was then extruded through a syringe, dried overnight (65°C), crumbled to reform pellets, and stored at -20°C until use (Control Diet). Fish were fed the Control Diet daily to satiation for 14 days at a set time to synchronize any associated behaviors. After 14 days, animals were acclimated to one of two diets; the Control Diet described above or a High-Magnesium Diet. The High-Magnesium Diet was created using the same procedure described above for the Control Diet, however during the formation of the paste using reverse osmosis water, 100mM MgCl_2_ was added. The pellets were reformed, dried, and stored as before. Animals were acclimated to the Control or High-Magnesium Diets for 21 days before sampling. For all treatments, fed animal were fed to satiation and sampled 3 hours post-meal ingestion. Additional animals acclimated to the diets were fasted for 7–10 days before sampling as unfed animals.

Fish were sampled following terminal anesthesia (buffered (pH = 7.5; Titrated with 1 N NaOH) tricaine methanesulfonate (MS-222; 0.25 g l^−1^; Western Chemical Inc, Ferndale, WA USA)). During sampling, a lateral incision was made along the body wall to expose the entire GIT (from esophagus to rectum), which was removed and placed into oxygenated Cortland saline (123mM NaCl, 5mM KCl, 1mM CaCl2, 1.9mM MgSO4, 11.9mM NaHCO3, 2.9mM NaH2PO4, 5.5m Glucose; pH = 7.4 (Titrated to the correct pH with 1N NaOH); 4°C) and kept on ice until use. The entire GIT was then sectioned into 8 equal lengths identified based on a proportion of total length. The sections were as follows: 1) esophagus, 2) anterior half of anterior gut (ant-ant), 3) posterior half of anterior gut (post-ant), 4) anterior half of mid gut (ant-mid), 5) posterior half of mid gut (post-mid), 6) anterior half of posterior gut (ant-post), 7) posterior half of posterior gut (post-post), and 8) rectum proceeding distally from the esophagus to the rectum.

### In vitro transport series

Following dissection, four experimental series were run as described below (Table 1). For non-everted tissue preps SIET measurements were obtained at the serosal surface and represent bulk transport within the serosal fluid. For these preparations, positive values indicate mucosal to serosal flux, whereas negative values indicate serosal to mucosal flux (Table 1). For everted preparations, SIET measurements were obtained at the mucosal surface and represent bulk transport in the mucosal fluid. For these preparations, positive values in indicate serosal to mucosal flux, whereas negative values indicate mucosal to serosal flux (Table 1). The Control Diet was used for all series, while the High-Magnesium Diet was used for Series 2 alone (Table 1).

**Table 1:** Summary of series preparations, saline compositions, and site of measurement.

Feeding Status and Diet	Preparation	Saline		Measurement	Direction of Flux
		Mucosal Saline	Serosal Saline		
**Series 1**					
Unfed	Non-everted	Cortland Saline	Cortland Saline	Serosal	+ve mucosal →serosal -ve serosal→mucosal
Fed Control Diet	Non-everted	Chyme	Cortland Saline	Serosal	+ve mucosal→serosal -ve serosal→mucosal
**Series 2**					
Unfed and Fed Control Diet	Non-everted	Cortland Saline	Cortland Saline	Serosal	+ve mucosal→serosal -ve serosal→mucosal
Unfed and Fed High -Magnesium Diet	Non-everted	Cortland Saline	Cortland Saline	Serosal	+ve mucosal→serosal -ve serosal→mucosal
**Series 3**					
Unfed	Non-everted	Cortland Saline + Magnesium	Cortland Saline	Serosal	+ve mucosal→serosal -ve serosal→mucosal
Unfed	Everted	Cortland Saline + Magnesium	Cortland Saline	Mucosal	+ve serosal-mucosal -ve mucosal→serosal
**Series 4**					
Unfed	Non-everted	Cortland Saline ± inhibitors	Cortland Saline ± inhibitors	Serosal	+ve mucosal→serosal -ve serosal→mucosal
Unfed	Everted	Cortland Saline ± inhibitors	Cortland Saline ± inhibitors	Mucosal	+ve serosal→mucosal -ve mucosal→serosal

See Materials and methods for Diet and Cortland Saline Concentration. +ve = positive, -ve = negative

For all series, non-everted, unfed preparations were filled with saline (Table 1) using a syringe and tied at both ends with silk ligatures, creating a sac. When preparing non-everted, fed intestinal tissues, similar sacs were created by either first flushing the chyme from the section with saline or by tying the ends of each section containing the chyme within (Table 1).

For all series, everted preparations were created by securing the esophagus or rectum section to a plastic pipette tip molten onto a thin metal wire (working very similar to a glass rod) and everting the tissue. The section was subsequently checked under a dissection microscope for tears and damage before use and filled as before (Table 1). Everted sacs made from fed animals were rinsed in Cortland saline to remove any remaining chyme from the mucosa.

Immediately before measurements, the GIT preparation under observation was placed into an agar-filled dish, pinned into place using fine metal insect pins on either side of the tissue, and immersed in a 20°C saline (Table 1) solution containing 2% 3.8mM MS-222 and 10 mM 4-(2-hydroxyethyl)-1-piperazineethanesulfonic acid (HEPES). MS-222 was added to the bath in order to minimize muscle contractions of the GIT while HEPES was added to saline used to measure proton (H^+^) fluxes alone for more stable readings.

#### Series 1. Zonation of net ion transport in unfed and fed animals

Net Mg^2+^, Ca^2+^, and H^+^ fluxes to (positive transport values) or from (negative transport values) the serosal fluid were determined for all non-everted intestinal sections (esophagus to rectum) from unfed and fed animals, revealing the location of highest net transport. For unfed animals (N = 4–5) transport within the intestinal sections was measured using symmetrical Cortland saline conditions (Table 1). For the fed animals (Control Diet; N = 4–7), transport within the intestinal sections was measured while containing chyme found within the sections (Table 1).

#### Series 2. Effect of dietary magnesium on ion transport rates

The esophagus and rectum were dissected from animals fed either the Control Diet or the High-Magnesium diet. Both non-everted and everted fed preparations were incubated with symmetrical salines placed on both the serosal and mucosal surfaces. Chyme was removed either by flushing non-everted preparations, or rinsing everted preparations in Cortland Saline. Mg^2+^, Ca^2+^, and H^+^ transport rates were measured at the mucosal surface in everted preparations (Control Diet N = 8; High-Magnesium Diet N = 8) and the serosal surface in non-everted preparations (Control Diet N = 8; High-Magnesium Diet N = 8).

#### Series 3. Ion transport kinetics

The esophagus and rectum were chosen for determination of Mg^2+^ transport kinetics, based on the highest net transport observed (Series 1). To determine Mg^2+^ transport kinetics, five mucosal saline Mg^2+^ concentrations were used to measure the impact on Mg^2+^, Ca^2+^ and H^+^ ion transport. Mg^2+^ concentrations in the mucosal saline were: 0, 1, 4, 16, 32, and 64 mM MgSO_4_ (made up in Cortland Saline). These Mg^2+^ concentrations were applied to the mucosal tissue, and transport from the mucosal lumen was measured directly as disappearance/appearance from the mucosal fluid in the everted preparations (N = 5), and indirectly as disappearance/appearance in the serosal media in the non-everted preparations (N = 4). The same tissue was used to measure transport at each concentrations and the order of concentrations used to fill the preps was randomized. Unfed tissues were used for all preparations. The Michaelis–Menten equation f = ax/(x + b), where f, transport rate; a = J max; b = Km and x = Mg^2+^ concentration was fit to the data where appropriate. Osmolarity of mucosal and serosal saline baths was balanced by the addition of a corresponding concentration of N-methyl-D-glucamine (meglumine; Sigma-Aldrich,St. Louis, MO, USA, pH = 7.4).

#### Series 4. Pharmacological impacts on transport

The impact of several transport inhibitors on Mg^2+^, Ca^2+^, and H^+^ transport were investigated. Esophageal (N = 6) and rectal (N = 6) tissues were once again chosen to measure net transport. The first inhibitor, Cobalt(III)-hexaammine chloride (Co3Hex; Sigma-Aldrich, 10^−3^ M) act as a Mg^2+^ channel inhibitor due to the radius of the molecule (244pm) closely mimicking that of the first hydration shell of Mg^2+^ (250pm; [34, 35]). The second inhibitor, ouabain (10^−4^ M; Sigma) has been extensively used as an inhibitor of active transport, due to its effect on Na^+^-K^+^-ATPase activity [2, 20]. Potential synergistic effects of Co3Hex and ouabain were tested by combining both inhibitors in the saline. The inhibitors were added to either the mucosal or serosal salines of both non-everted and everted preparations (Table 1) creating 4 treatments: Everted tissues with inhibitors in the mucosal bath and measurements at the mucosal surface or inhibitors within the serosal fluid and measurements at the mucosal surface; Non-everted tissues with inhibitors in the serosal bath and measurements at the serosal surface or inhibitors within the mucosal fluid and measurements at the serosal surface. Each inhibitor was applied the same tissue preparation in a random order. Application consisted of bathing the tissue in Cortland saline containing the inhibitor for 10 minutes, followed by measurement of transport while exposed. Between inhibitor applications, the tissues were bathed in Cortland saline for 10 minute to allow recovery of transport. Recovery of transport was quantified before application of subsequent inhibitors.

### Scanning Ion-selective Electrode Technique (SIET)

The SIET technique was used to measure ion flux to the bath surrounding preparations as previously described [26, 29]. An ion-selective microelectrode (ISME) was mounted on and controlled by a 3D micro-stepper motor manipulator (CMC-4; Applicable Electronics, Forestdale, MA) as programmed by the automated scanning electrode technique software (ASET; Sciencewares, East Falmouth, MA, USA). Voltage gradients (_Δ_V in mV) were measured between the intestinal surface and a set distance away from the tissue, which were ultimately used to calculate the ion flux (see the calculations section). Both the ISME and a reference electrode were connected to a headstage (by an Ag/AgCl wire holder for the former and Ag/AgCl half-cell for the latter; WPI, Sarasota, FL), which was in turn connected to an ion polarographic amplifier (IPA-2; Applicable Electronics, Forestdale, MA).

All reference electrodes were constructed as outlined in [29]. Briefly, a borosilicate glass microcapillary (model TW150-4; WPI) was heated at one end to form a 45° bend and filled with 3M KCl solution containing 3% agar. The solution was allowed to harden, and the references electrodes were stored in 3 M KCl between uses.

To form microelectrodes, glass capillaries (model TW150-4; WPI, Sarasota, FL) were pulled on a P-97 Flaming-Brown horizontal micropipette puller (Sutter Instruments, Novato, CA) to a tip diameter of 5–8μm as described in [29]. The microelectrodes were then heated to 350°C for 15 minutes and subsequently vapour silanized by covering with a borosilicate dish containing N,N-dimethyltrimethylsilylamine (Fluka, Buchs, Switzerland; ~1μl per electrode. Silanization proceeded for 1 hr at 350°C, after which the microelectrodes were cooled and stored until further use. The microelectrodes were re-silanized every 14 days in order to maintain effective silanization. ISMEs were constructed immediately before each use. Briefly, the bore of silanized microelectrodes were filled with an appropriate backfill. The selected ionophore was then added to the tip by front-filling the microelectrode for a column length of 100-150nm. Finally, the Mg^2+^ and Ca^2+^ selective microelectrodes were briefly dipped in tetrahydrofuran-dissolved polyvinyl chloride (PVC; Fluka, Buchs, Switzerland) as described by [36]. This prevented loss of the ionophore during measurement. The Mg^2+^-selective microelectrodes were constructed with the Magnesium ionophore II (Fluka Chemical Co., Ronkonkoma, NY, USA) and were backfilled with 100mM MgCl_2_. Mg^2+^ microelectrodes were then calibrated using 1mM and 10mM MgCl_2_ solutions, obtaining an average slope of 29.741 ± 0.254 (mean ± SEM) during calibration. Because of the potential affinity of the Mg^2+^ ionophore II for Ca^2+^ and H^+^, all three ions were concurrently measured to correct for overestimation of Mg^2+^ movement. The Ca^2+^-selective microelectrodes were constructed with Calcium ionophore II cocktail (Fluka Chemical Co., Ronkonkoma, NY, USA) and backfilled with a 100mM CaCl_2_ solution. The calibration solutions used were 1mM and 10mM CaCl_2_ solutions, producing an average slope of 31 ± 1.2. Finally, the H^+^ microelectrodes were constructed with H^+^ Ionophore I Cocktail B (SigmaAldrich, ON, CA) with a 100mM NaCl, 100 mM sodium citrate backfill (buffered to pH 6.0). These microelectrodes were not dipped in PVC as it would interfere and prevent near-Nernstian slope from being achieved. The H^+^ electrodes were calibrated using pH7 and pH10 calibration solutions (Sartorius Stedim North America Inc., Bohemia, NY, USA) giving a slope of 52.862 ± 0.421. When the H^+^-selective microelectrode was being used, 10mM of HEPES was added to the mucosal and saline baths for more stable readings. Both the Ca^2+^ and H^+^ ionophores have excellent selectivity for their respective ions and do not require additional measurements.

To ensure the slope generated during Mg^2+^ measurement was not impacted by interference from Ca^2+^ or H^+^, the effect of various Ca^2+^ (0.5 mM– 5 mM) and H^+^ (pH 7.0–8.0) bath concentrations on the voltage detected using the Mg^2+^ ionophore II when measuring Mg^2+^ within solutions was tested. Interference of this nature would prevent conclusions about Mg^2+^ transport, however no interference was detected at these experimental concentrations. Knowing the slope of the Mg^2+^ electrode was not impacted, when changes in Mg^2+^ flux were detected and not mirrored by changes in Ca^2+^ and H^+^ flux, we concluded Mg^2+^ transport alterations were present alone (i.e. if Mg^2+^ transport rates increased but Ca^2+^ and H^+^ transport remained constant, then only changes in Mg^2+^ transport were observed). When Mg^2+^ transport changes were mirrored by alterations in Ca^2+^ and/or H^+^ transport, we could not conclude that alterations in transport rates were due to alterations in Mg^2+^ alone (i.e. if Mg^2+^ transport rates increased as did Ca^2+^, then we cannot infer that Mg^2+^ transport was changing as the changes may be attributable to Ca^2+^).

### Corroborating SIET Mg^2+^ values

In a separate experiment, Series 1 and Series 3 were repeated however Mg^2+^ transport was measured using ISMEs constructed using Magnesium ionophore VI (Fluka Chemical Co., Ronkonkoma, NY, USA), again backfilled with 100mM MgCl_2_. The Magnesium ionophore VI cocktail was prepared in lab according to [37, 38]. Briefly, 1% weight of powdered ionophore was mixed with a lipophilic salt (potassium tetrakis (4-chlorophenyl)borate) at a molar ratio of 150% relative to ionophore [37]. The remainder was the solvent 2-nitrophenyl octyl ether [37]. This cocktail was used to create ISMEs that were then dipped in PVC before use. Without PVC, the electrodes would not function [38]. This method of preparation was compared to that of [37] where PVC was dissolved in tetrahydrofuran and incorporated at 33% weight to the cocktail mix. Due to a lack of detectable differences between the methods for Mg^2+^ detection, and the increased ease of dipping the ISME into PVC, this method of preparation was chosen for all experiments. The ISME were calibrated using 1mM and 10mM MgCl_2_ solutions as before, obtaining an average slope of 30.102 ± 0.198 (mean ± SEM) during calibration. Magnesium ionophore VI has a far greater selectivity for Mg^2+^ over Ca^2+^ and H^+^, reducing interference from these ions.

### Concentration gradient and flux calculations

The voltage gradient ASET readings were converted into ionic concentration gradients with the following formula (described in [27, 36]:

$$\Delta C={\text{C}}_{\text{B}}\times 10^{\left(\frac{\Delta V}{S}\right)}-{\text{C}}_{\text{B}}$$ (1)

where ΔC is the concentration gradient between the “at” and “away” points (calculated in μmol l^-1^ cm^-3^), C_B_ is the background ion concentration (recorded in μmol l^-1^), ΔV is the voltage gradient (μV) and S is the slope of the electrode over a 10-fold difference in ion concentration.

Ultimately, ΔC can be converted into flux using Fick’s law of diffusion:

$$J_{I}=D_{I}\times \Delta C÷\Delta X$$ (2)

where J_I_ is the net flux (measured in pmol cm^-2^ s^-1^), D_I_ is the diffusion coefficient of the measured ion (1.19x10^-5^ cm^2^ s^-1^ for Ca^2+^; 7.1x10^-6^ cm^2^ s^-1^ for Mg^2+^; 9.4x10^-5^ cm^2^ s^-1^ for H^+^) and ΔX is the distance between the two points measured in cm.

Proton measurements were adjusted for buffering capacity of the solution (as described in [39]) with the following equations:

$$J_{Htotal}=J_{I}\times \left(1+x_{i}+\cdots x_{n}\right)$$ (3)

And

$$x_{i}=\frac{D_{B}}{D_{H^{+}}}\times \left[B\right]\times \frac{K_{a}}{{\left(K_{a}+\left[H^{+}\right]\right)}^{2}}$$ (4)

where D_B_ is the diffusion coefficient of the individual buffers present in the solution (HEPES: 6.2x10^-6^ cm^2^ s^-1^; bicarbonate: 1.2 x10^-5^ cm^2^ s^-1^; sulphate: 5.0 x10^-6^ cm^2^ s^-1^; phosphate: 3.6 x10^-6^ cm^2^ s^-1^), D_H_^+^ is the diffusion coefficient for H^+^, [B] is the concentration of the individual buffer, K_a_ is the dissociation constant for the individual buffer, and [H^+^] is the concentration of the proton. The diffusion coefficient for MS-222 is not known, however as it was present at such low concentrations the impact of correction for the buffer is likely minimal.

### Statistics

All statistical tests were carried out in SigmaStat 3 and plots constructed in SigmaPlot11 (Systat). Before running parametric tests, data was first examined for normality and homogeneity of variance. Zonation of transport and impact of feeding was examined using a repeated measures two-way ANOVA (with section and feeding as factors). The impact of dietary Mg^2+^ on ion transport rates was examined with a repeated measures two-way ANOVA (with section and feeding status as factors). Kinetic data was modelled using Sigmaplot for line of best fit with linear regression or Michaelis–Menten kinetics with single site saturation. The impact of inhibitors on the relative ion transport within each section was examined using a one-way repeated-measures ANOVA (inhibitor as factor). All were followed by a Holm-Sidak post-hoc test. Significance was assessed at p<0.05. Values are presented as mean ± S.E. (N = individual preparations).

## Results

### Intestinal zonation and impact of feeding

**Fig 1:**
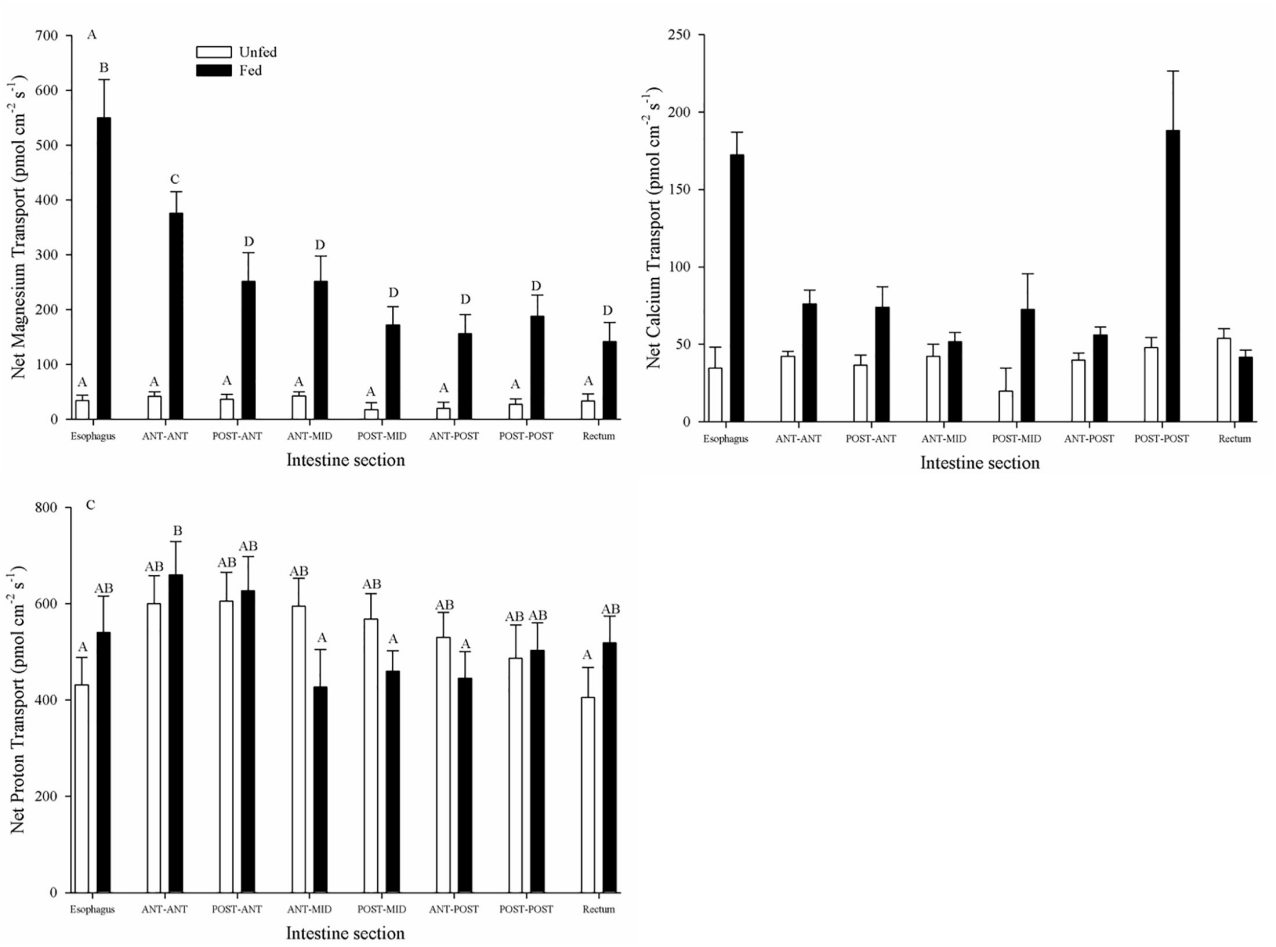
Zonation of intestinal ion transport in unfed and fed *C*. *auratus*. A) Mg^2+^ flux zonation along the GIT for both fed (N = 5) and fasted (N = 7) *C*. *auratus*. B) Ca^2+^ flux zonation along the GIT for both fed (N = 4) and fasted (N = 4) *C*. *auratus*. C) H^+^ flux zonation along the GIT for both fed (N = 4) and fasted (N = 4) *C*. *auratus*. Non-everted preparations were created by dividing the GIT into 8 equal length segments. Positive numbers indicate appearance in the serosal fluid suggesting mucosal-to-serosal movement. Data represent mean flux ± S.E.M. A two-way repeated measures ANOVA followed by a Holm-Sidak test was used (segment and fasting status as factors). No significant interaction effect was detected. Different letters within each panel indicate significant difference (p<0.05).

Mg^2+^ transport along the unfed GIT was similar in all sections, averaging an appearance in the serosal fluid rate of 29.9 ± 7.2 pmol cm^-2^ s^-1^ (N = 7) across all segments (Fig 1A). Mg^2+^ appearance was significantly higher in all chyme-containing sections from fed fish, relative to unfed fish, increasing between 2–5 fold (Fig 1A). Furthermore, Mg^2+^ appearance was generally higher in the anterior segments and lower in the posterior segments in fed fish. In particular, with the esophagus (551.2 ± 70.3 pmol cm^-2^ s^-1^) showing a significantly higher appearance rate, decreasing 2 fold to 252.8 ± 52.2 pmol cm^-2^ s^-1^ (N = 7) in the post-ant, and thereafter remaining unchanged for an average transport rate of 167.0 ± 21.7 pmol cm^-2^ s^-1^ (N = 7) across all remaining segments (Fig 1A). Ca^2+^ displayed several similar trends compared to Mg^2+^. Unfed transport rates remained significantly unchanged across all sections (average across all sections: 47.6 ± 5.1 pmol cm^-2^ s^-1^ (N = 4)) and fed appearance rates generally exceeded unfed appearance rates in most chyme-containing sections (Fig 1B). However, while the fed esophagus Ca^2+^ transport rates (172±14 pmol cm^-2^ s^-1^(N = 4)) were significantly higher than the fed mid sections (ranging from 51 ± 6 pmol cm^-2^ s^-1^ at the post-mid to 75±8.9 pmol cm^-2^ s^-1^ at the ant-ant) as with Mg^2+^, in contrast fed Ca^2+^ appearance rates recovered in the post-post (187±38 pmol cm^-2^ s^-1^ (N = 4)) segment, before decreasing again in the rectum (Fig 1B). The pattern of H^+^ flux zonation was dissimilar from either of the divalent ions (Fig 1C). The H^+^ transport rate and direction at the anterior (esophagus, ant-ant, post-ant), mid (ant-mid and post-mid), and posterior (ant-post, post-post and rectum) GIT segments was not significantly different between unfed and fed animals despite fed sections containing chyme (Fig 1C). Unfed animals showed no significant differences along the GIT, averaging 521 ± 22 pmol cm^-2^ s^-1^ across all sections (N = 4; Fig 1C). The fed ant-ant (659±77 pmol cm^-2^ s^-1^) and post-ant (627±71 pmol cm^-2^ s^-1^) segments transport rates were significantly elevated from the fed ant-mid (427±82 pmol cm^-2^ s^-1^), post-mid (459±79 pmol cm^-2^ s^-1^), ant-post (445±85 pmol cm^-2^ s^-1^) segments (N = 4; Fig 1C).

### Impact of dietary Mg^2+^ on ion transport rates

**Fig 2:**
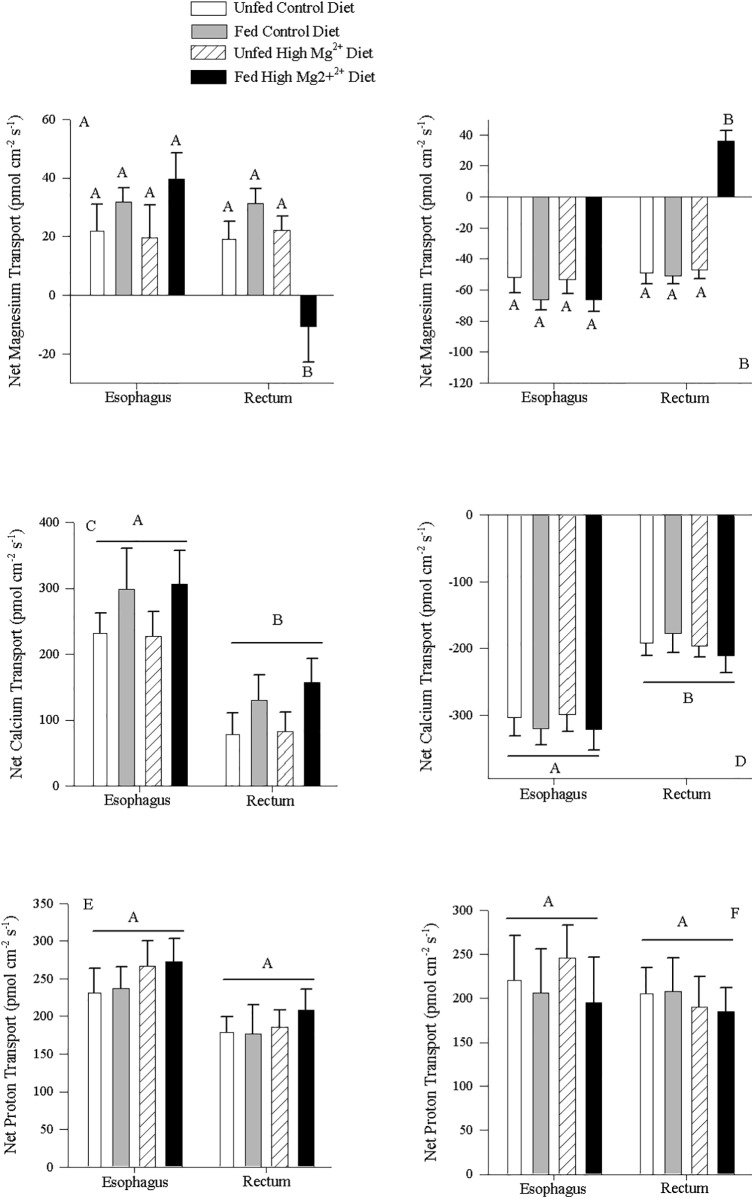
Dietary magnesium impact on intestinal ion transport by esophageal and rectal tissues. A) Mg^2+^ C) Ca^2+^ and E) H^+^ transport in the GIT for fed *C*. *auratus* consuming either the Control Diet (N = 8) or High-Magnesium Diet (N = 8) in non-everted preparations with symmetrical Cortland saline for mucosal and serosal compartments. Positive values indicate appearance of ion in the serosal fluid suggesting mucosal-to-serosal movement, Negative values indicate disappearance of ion in the serosal fluid suggesting serosal-to-mucosal movement. B) Mg^2+^ D) Ca^2+^ and F) H^+^ transport in the GIT for fed *C*. *auratus* consuming either the Control Diet (N = 8) or High-Magnesium Diet (N = 8) in everted preparations with symmetrical Cortland saline for mucosal and serosal compartments. Negative values indicate disappearance of ion in the mucosal fluid suggesting mucosal-to-serosal movement. Positive values indicate appearance of ion in the mucosal fluid suggesting serosal-to-mucosal movement, Data represent mean flux ± S.E.M. A two-way repeated measures ANOVA followed by a Holm-Sidak test was used (segment and diet as factors). No significant interaction effect was detected. Different letters within each panel indicate a significant difference (p<0.05).

Under symmetrical conditions, consuming the control diet did not significantly increase the rate of Mg^2+^ transport in the esophagus or the rectum in non-everted (Fig 2A) and everted tissues (Fig 2B) over unfed values. Most values were positive for non-everted tissues (Fig 2A) and negative for everted tissues (Fig 2B), indicating appearance of Mg^2+^ in the serosal fluid and disappearance from the mucosal fluid, suggesting absorption. However, consuming the High-Magnesium Diet resulted in a disappearance of Mg^2+^ from the serosal fluid in the non-everted rectal tissue (Fig 2A) and an appearance of Mg^2+^ in the mucosal fluid of everted tissue (Fig 2B) suggesting secretion. Dietary Mg^2+^ and feeding status did not alter the transport rate of Ca^2+^ across the tissues of either the esophagus or the rectum for both non-everted (Fig 2C) and everted (Fig 2D) tissues. However, the esophageal tissues showed an average higher rate of appearance in the serosal fluid across treatments (265.2 ± 32.1 pmol cm^-2^ s^-1^ (N = 16) than the rectal tissues (122.7 ± 24.5 pmol cm^-2^ s^-1^ (N = 16); Fig 2C). This was consistent with the everted tissues, that showed an average higher rate of disappearance of Ca^2+^ from the mucosal fluid by the esophageal tissue (-312.6 ± 18.7 pmol cm^-2^ s^-1^ (N = 16)) compared to the rectal tissue (-195.3 ± 18.7 pmol cm^-2^ s^-1^ (N = 16); Fig 2D). Dietary Mg^2+^ content and feeding status did not affect the net transport of H^+^ into the serosal fluid, and both the non-everted esophagus and rectal tissues showed similar appearance rates averaging across treatments at 219.9 ± 13.9 pmol cm^-2^ s^-1^ (N = 16; Fig 2E). Similarly, the everted tissue preparations were not affected by feeding status or diet, however these tissues also displayed appearance in the mucosal fluid, averaging across treatments at 193.8 ± 13.9 pmol cm^-2^ s^-1^ (N = 16; Fig 2E).

### Ion transport kinetics with increasing luminal Mg^2+^ concentration

**Fig 3:**
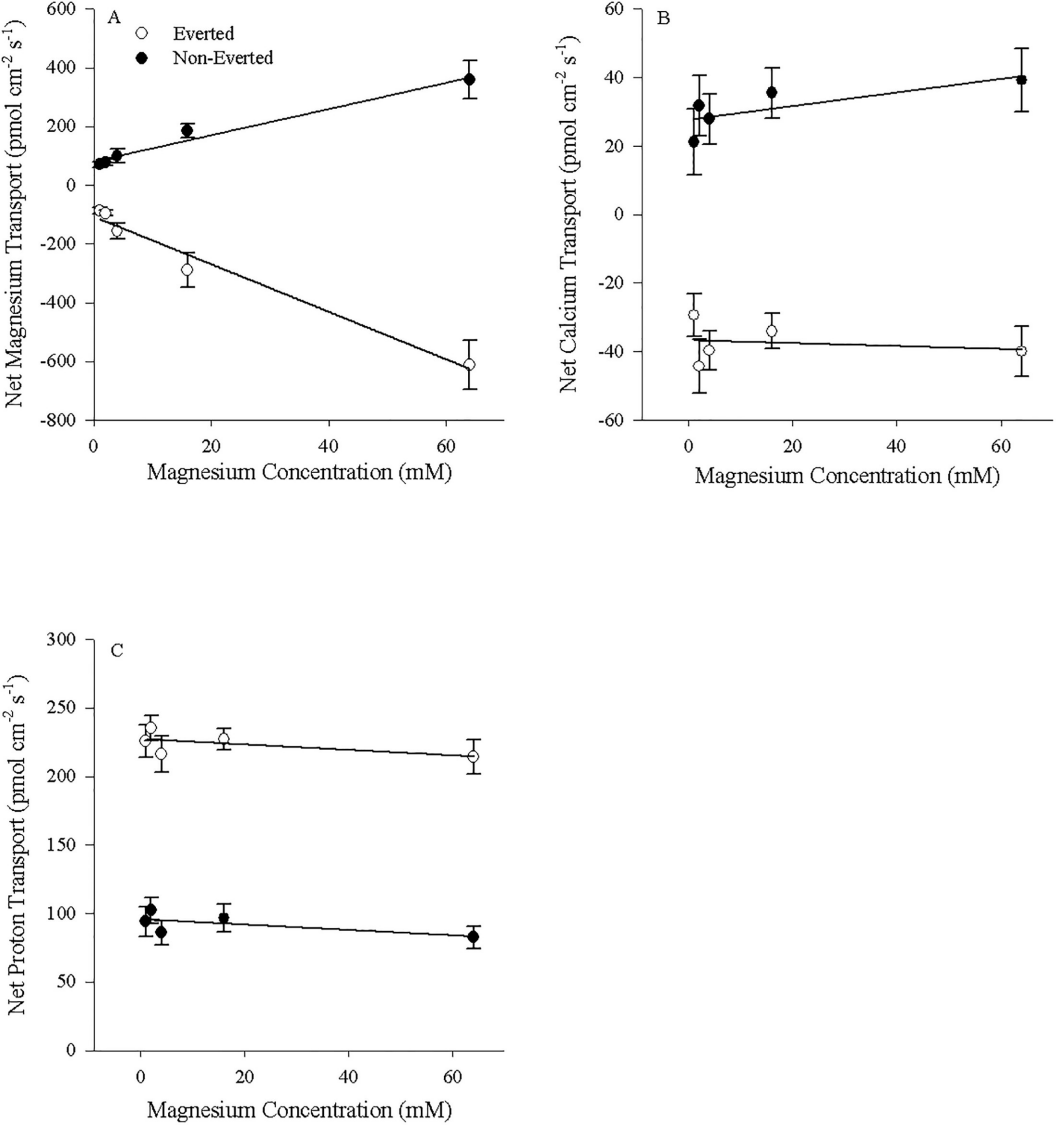
Esophageal transport kinetics by both everted and non-everted unfed tissues. A) Mg^2+^, B) Ca^2+^, and C) H^+^ fluxes in the serosal fluid for non-everted (N = 4) and the mucosal fluid for everted (N = 5) esophageal tissue of unfed *C*. *auratus*. Cortland saline containing 0–64 mM MgSO_4_ was applied to the mucosal surface. Positive values in non-everted preparations indicate appearance in the serosal fluid (suggesting mucosal-to-serosal movement) and negative values in everted preparations indicate disappearance from the mucosal fluid (suggesting mucosal-to-serosal movement). Data represent mean flux ± S.E.M. Regression analysis revealed a significant (p<0.05) relationship between Mg^2+^ concentration and Mg^2+^ transport rate. No significant relationship between Mg^2+^ concentration and Ca^2+^ or H^+^ transport rates was found. See text for more details.

Mg^2+^ transport into the serosal fluid by non-everted esophageal preparations (positive values) and out of the mucosal fluid by everted preparations (negative values) correlated linearly with alterations in the mucosal concentrations of Mg^2+^ (y = 4.45x + 80.9, (R^2^ = 0.983) and y = -8.09x − 107.6 (R^2^ = 0.975) respectively; p< 0.05; Fig 3A). Ca^2+^ transport rates in both preparations were not significantly correlated with increased luminal Mg^2+^ concentration (p>0.05; non-everted: y = 0.19x + 27.7 (R^2^ = 0.476); everted: y = -0.04x − 36.7 (R^2^ = 0.043); Fig 3B). Likewise, H^+^ transport rates in both preparations were not significantly correlated with increased luminal Mg^2+^ concentration (p>0.05; non-everted: y = -0.196x + 95.7 (R^2^ = 0.429); everted: y = -0.199x + 227.2 (R^2^ = 0.357); Fig 3C).

**Fig 4:**
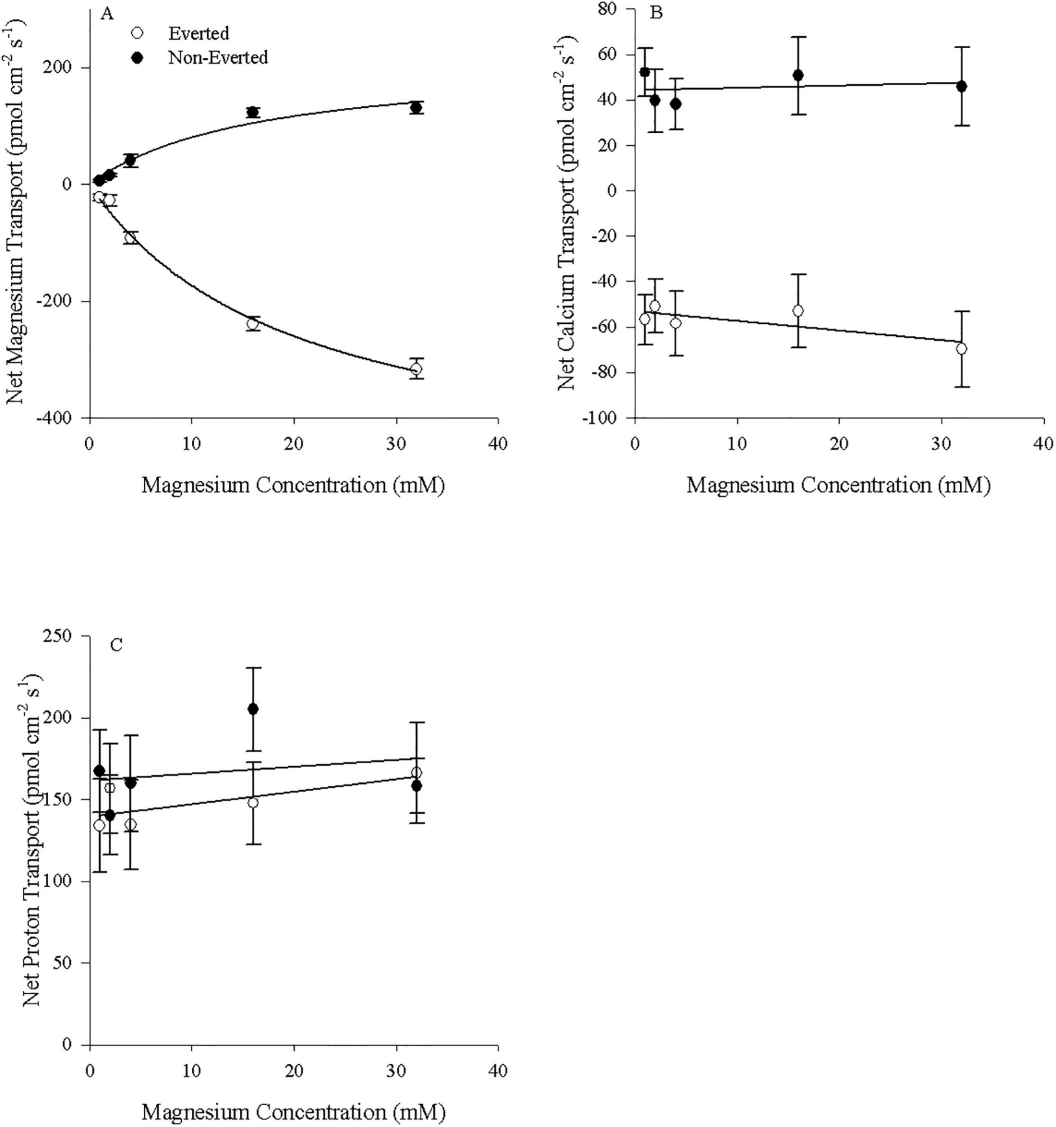
Rectal tissue transport kinetics by both everted and non-everted unfed tissues. A) Mg^2+^, B) Ca^2+^, and C) H^+^ fluxes in the serosal fluid for non-everted (N = 4) and the mucosal fluid for everted (N = 5) rectal tissue of unfed *C*. *auratus*. Cortland saline containing 0–32 mM MgSO_4_ was applied to the mucosal surface. Positive values in non-everted preparations indicate appearance in the serosal fluid (suggesting mucosal-to-serosal movement) and negative values in everted preparations indicate disappearance from the mucosal fluid (suggesting mucosal-to-serosal movement). Data represent the mean flux ± S.E.M. Regression analysis revealed a significant (p < 0.05) relationship between Mg^2+^ concentration and Mg^2+^ transport rate fitting a Michaelis-Menten curve with single saturation. No significant relationship between Mg^2+^ concentration and Ca^2+^ or H^+^ transport rates was found. See text for more details.

The relationship between Mg^2+^ transport and Mg^2+^ concentration within the non-everted rectal tissue preparations was defined by the Michaelis–Menten equation f = 211.7.7x/(16.7 + x) (R^2^ = 0.989; Fig 4A) with Mg appearing in the serosal fluid. For the everted preparations, following conversion to positive values (as transport indicated disappearance from the mucosal fluid), the Michaelis–Menten equation f = 520.1x/(19.9 + x) similarly defined the correlation between Mg^2+^ transport and Mg^2+^ concentration (R^2^ = 0.997; Fig 4A). As seen with the esophageal tissue, Ca^2+^ fluxes in both rectal tissue preparations were not significantly correlated with increased luminal Mg^2+^ concentration (p>0.05; non-everted: y = 0.01x + 44.7 (R^2^ = 0.017); everted: y = -0.424x − 53.7 (R^2^ = 0.344); Fig 4B). Finally, H^+^ fluxes in both preparations were also not significantly correlated with increased luminal Mg^2+^ concentration (p>0.05; non-everted: y = 0.4x + 161.4 (R^2^ = 0.053); everted: y = 0.7x + 139.4 (R^2^ = 0.497); Fig 4C).

### Inhibition of ion transport in non-everted esophagi and rectums

**Fig 5:**
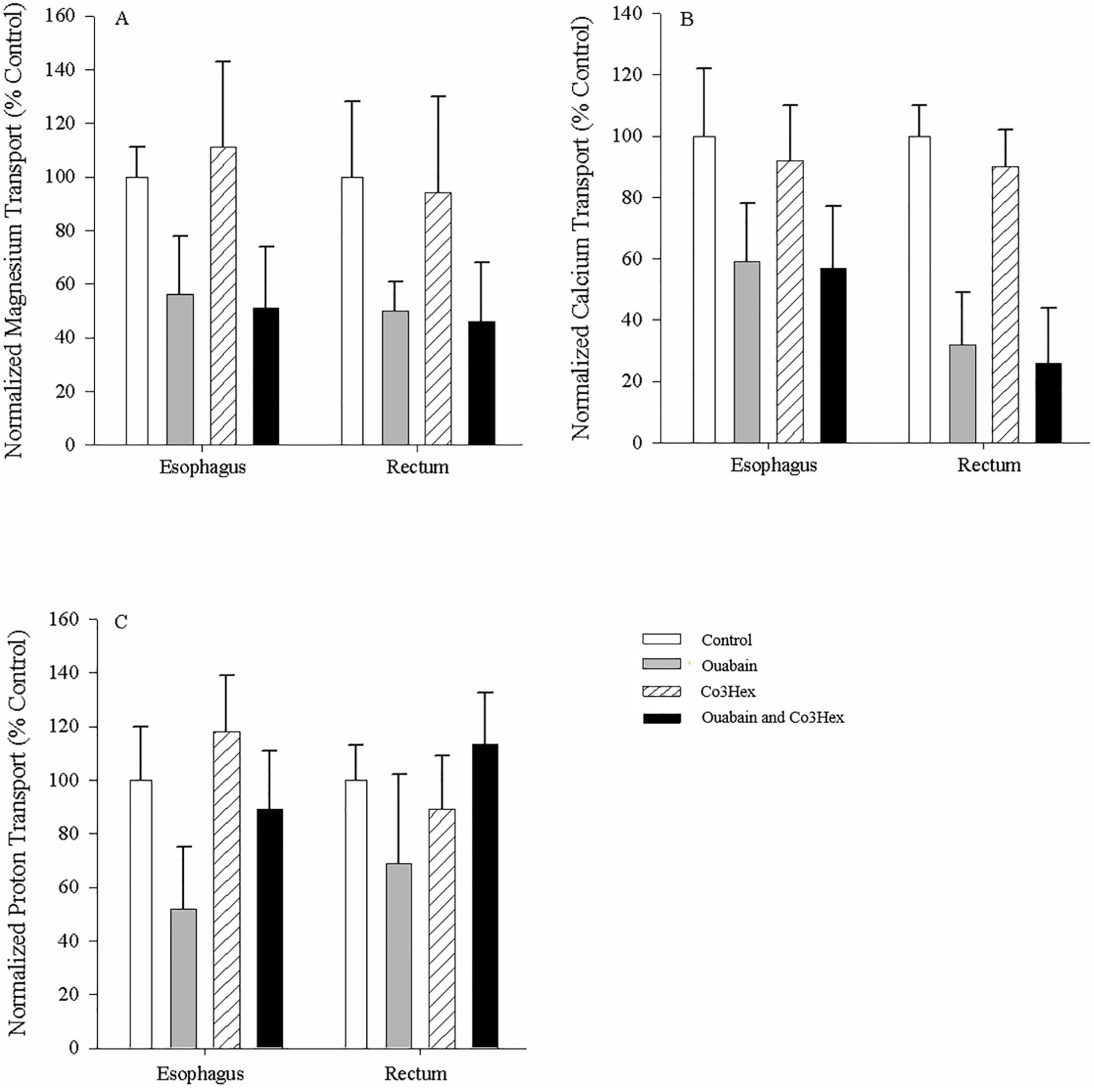
Serosal esophagus and rectal ion flux in response to serosal application of inhibitors. Effect of application of Cortland’s saline containing 0.1mM ouabain, 0.1mM Co3Hex or both inhibitors to the serosal surface of non-everted esophageal and rectal unfed *C*. *auratus* tissues on relative Mg^2+^ (A), Ca^2+^ (B) and H^+^ (C) transport. Values are relative to the control treatment (symmetrical application of Cortland saline). The salines used on both surfaces of the tissues were buffered with 10mM HEPES (pH = 7.4). All measurements were taken at the serosal surface. Data are mean flux ± S.E.M. N = 6. A repeated measures one-way ANOVA (inhibitor as factor) and post-hoc Holm-Sidak test was used to compare individual treatments to the control (* indicates significant difference (p<0.05) from control).

**Fig 6:**
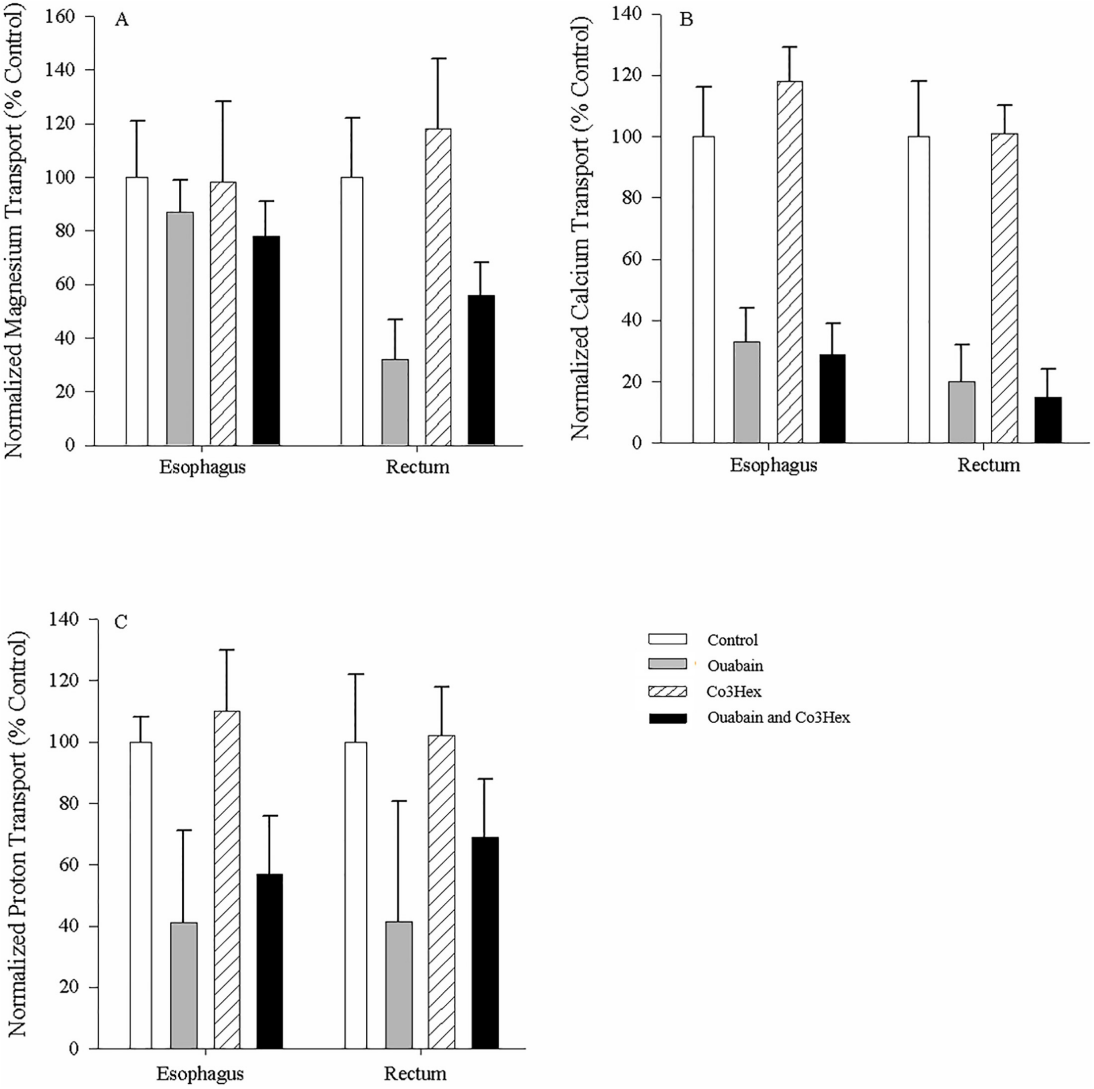
Serosal esophagus and rectal ion flux in response to mucosal application of inhibitors. Effect of application of Cortland’s saline containing 0.1mM ouabain, 0.1mM Co3Hex or both inhibitors to the mucosal surface of non-everted esophageal and rectal unfed *C*. *auratus* tissue on relative Mg^2+^ (A), Ca^2+^ (B) and H^+^ (C) transport. Values are relative to the control treatment (symmetrical application of Cortland saline). Salines used on both surfaces were buffered with 10mM HEPES (pH = 7.4). All measurements were taken at the serosal surface. Data are mean flux ± S.E.M. N = 6. A repeated measures one-way ANOVA (inhibitor as factor) and post-hoc Holm-Sidak test was used to compare individual treatments to the control (* indicates significant difference (p<0.05) from control).

All fluxes for the divalent ions as well as protons in all of the non-everted tissue preparations were positive, indicative of ion transport into the serosal fluid, or secretion (Figs 5 and 6). Serosal application of individual and combined inhibitors was not effective at altering esophageal transport (Fig 5A). Application of ouabain alone to the serosal surface significantly reduced Mg^2+^ fluxes (to 49.9 ± 11.1% of control (N = 6)) in the rectal tissue (Fig 5A), while application of both ouabain and Co3Hex was suggestive of inhibition (p = 0.061). In comparison, esophageal Ca^2+^ fluxes were likewise unaffected by inhibitor application, while rectal Ca^2+^ fluxes were significantly reduced when ouabain or ouabain + Co3Hex was applied to the serosal surface (32.2 ± 16.8 and 26.0 ± 18.0% respectively (N = 6); Fig 5B). Serosal application of individual and combined inhibitors was not effective at altering H^+^ transport in either tissue (Fig 5C).

Mucosal application of inhibitors once again failed to alter Mg^2+^ transport in the esophageal tissue (Fig 6A). As well, mucosal application of ouabain was again able to reduce Mg^2+^ flux rate (32.4 ± 14.9% of control (N = 6); Fig 6A). A novel significant reduction in Mg^2+^ flux rates (56.5 ± 12.1% of control (N = 6)) was observed with mucosal application of ouabain + Co3Hex (Fig 6A). Mucosal application of ouabain and ouabain + Co3Hex likewise decreased Ca^2+^ fluxes in the rectal tissue (20.3 ± 12.2 and 15.1 ± 8.8% respectively (N = 6); Fig 6B) as seen with the serosal application. A novel reduction in Ca^2+^ fluxes in the espohageal tissue was apparent with the mucosal application of ouabain (32.9 ± 10.9% (N = 6)) and ouabain + Co3Hex (29.2 ± 10.0% (N = 6); Fig 6B). Mucosal application of the inhibitors also did not affect H^+^ fluxes in the esophageal and rectal tissues (Fig 6C) as seen with serosal applications. For all tissue preparations, Co3Hex alone had no impact on ion transport (Figs 5 and 6) suggesting the decrease when both ouabain and Co3Hex inhibitors were applied was due to ouabain alone.

### Inhibition of ion fluxes in everted rectal tissue

**Fig 7:**
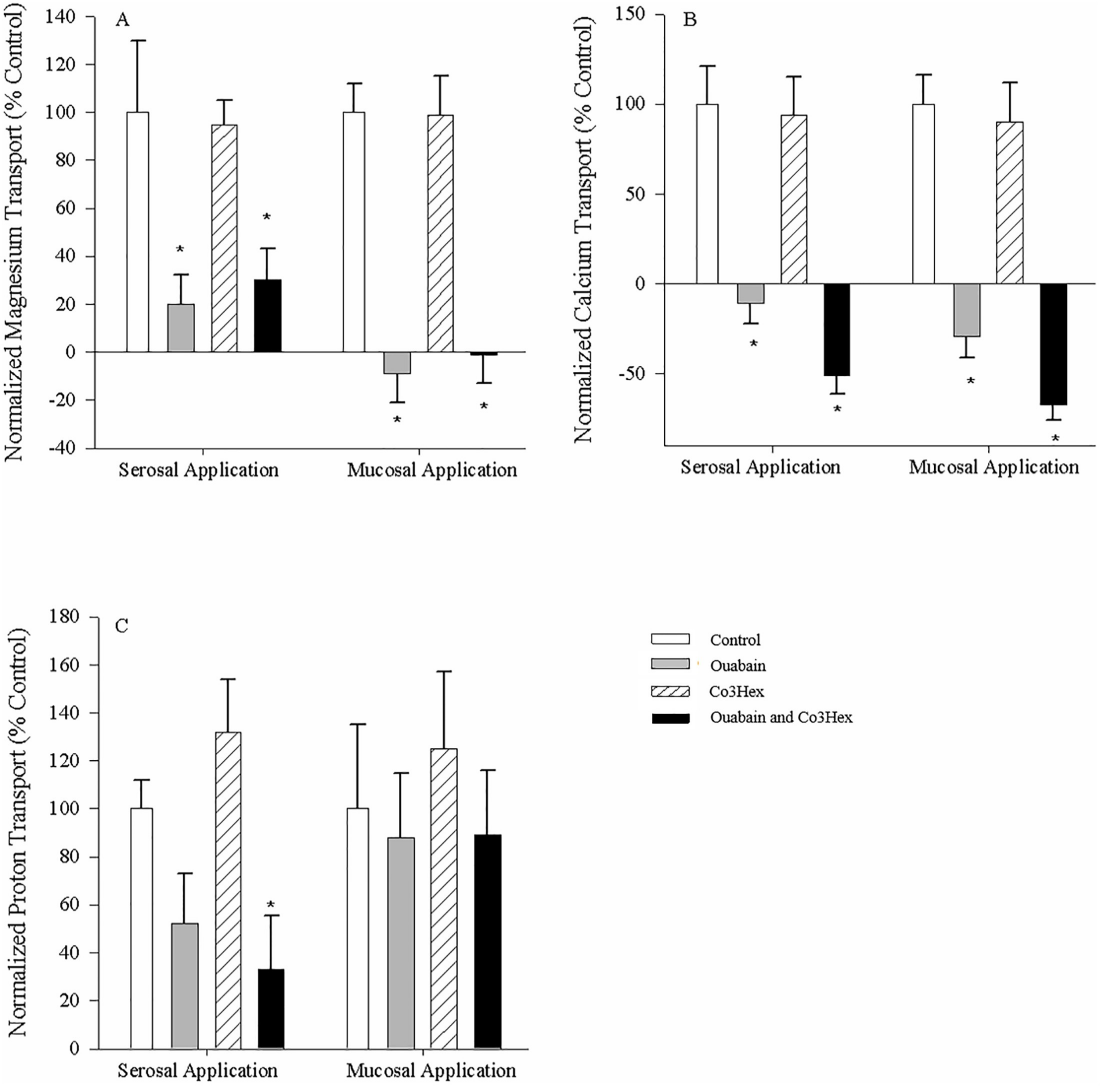
Mucosal rectal ion flux in response to serosal and mucosal application of inhibitors to everted tissues. Mucosal Mg^2+^ (A), Ca^2+^ (B) and H^+^ (C) flux in response to serosal and mucosal application of Cortland saline containing 0.1mM ouabain, 0.1mM Co3Hex or both inhibitors to the everted esophagus from unfed *C*. *auratus*. Values are relative to the control treatment (symmetrical application of Cortland saline). Salines used on both surfaces were buffered with 10mM HEPES (pH = 7.4). All measurements were taken at the mucosal surface. Data are mean flux ± S.E.M. N = 7. A repeated measures one-way ANOVA (inhibitor as factor) and post-hoc Holm-Sidak test was used to compare individual treatments to the control (* indicates significant difference (p<0.05) from control).

The Mg^2+^ and Ca^2+^ fluxes for control, everted tissues were negative, indicating disappearance from the mucosal fluid or absorption, while the H^+^ fluxes were positive indicating appearance or secretion (Fig 7). Serosal application of ouabain alone or ouabain + Co3Hex reduced Mg^2+^ flux in the tissue to 20.5 ± 12.7% and 30.2 ± 13.3% of control values respectively (N = 7; Fig 7A), a greater effect than seen with non-everted preparations. Likewise, ouabain additionally decreased and reversed Mg^2+^ flux when applied to the mucosal surface either alone (-9.2 ± 11.8%) or in combination with Co3Hex (-1.3 ± 12.1% (N = 7); Fig 7A). Ca^2+^ fluxes were similarly reversed in preparations containing ouabain, from absorption to secretion when applied to the serosal and mucosal salines of the everted tissues (-10.9 ± 10.7% and -51.2 ± 9.8% of control respectively (N = 7); Fig 7B), suggesting a similar but exaggerated impact as when applied to non-everted tissues (Figs 5B and 6B). Furthermore, Ca^2+^ fluxes were also reversed when both inhibitors were present, reversing to -28.8 ± 12.1% (N = 7) when applied to the serosal surface and -67.2 ± 9.3% (N = 7) when applied to the mucosal surface (Fig 7B). H^+^ fluxes decreased with ouabain + Co3Hex (to 33.3 ± 25.7% of control (N = 7)) when applied to the serosal surface of the everted tissues, but not the mucosal surface (Fig 7C). Mucosal applications of any inhibitor failed to elicit as response (Fig 7C). Once again, Co3Hex failed to induce significant changes in any of the fluxes in everted rectum, regardless of the surface it was applied to (Fig 7).

### Corroboration of Mg^2+^ transport using magnesium ionophore VI

**Fig 8:**
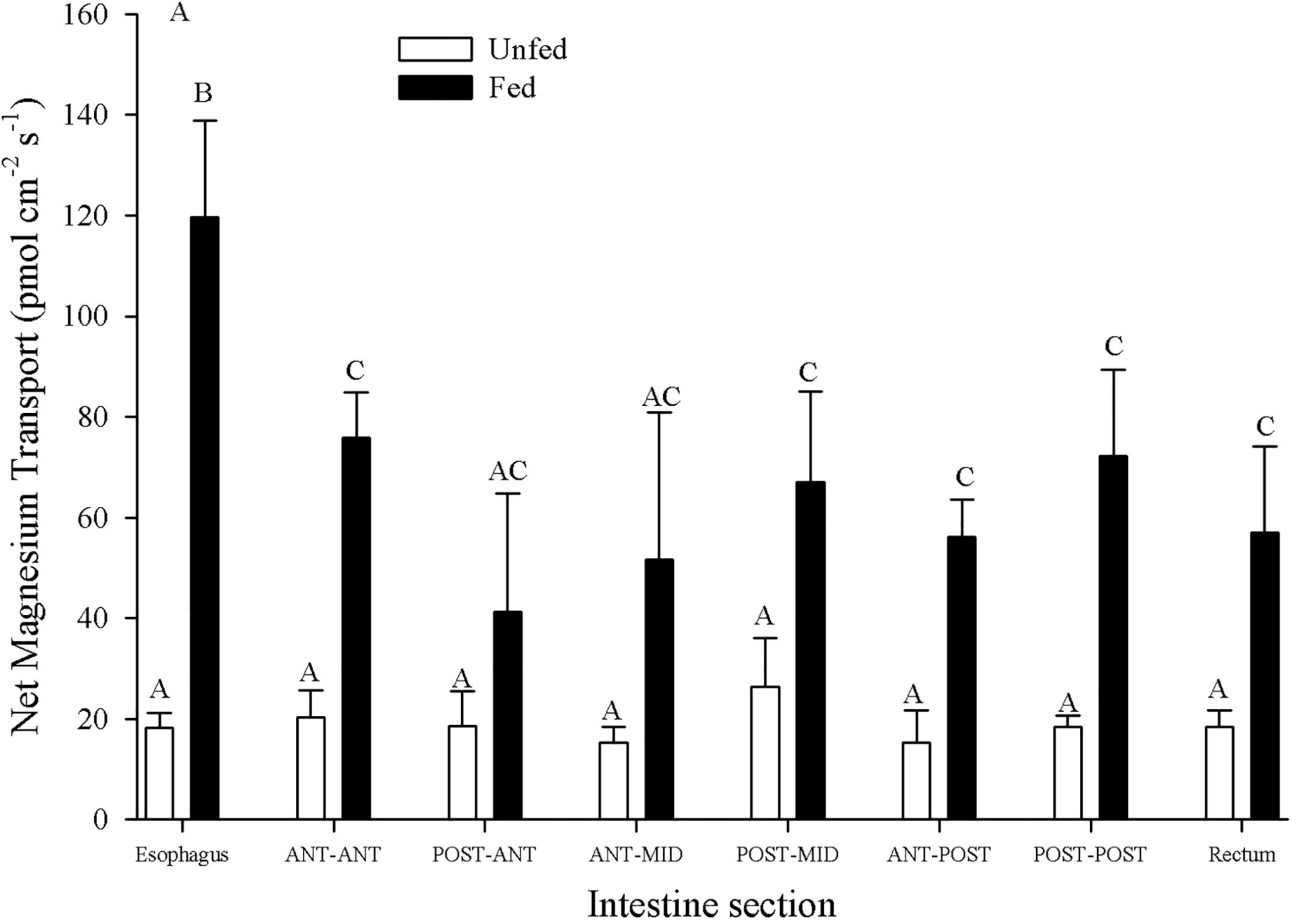
Zonation of intestinal ion transport in unfed and fed *C*. *auratus* measured with magnesium ionophore VI. Mg^2+^ flux zonation along the GIT for both fed (N = 6) and fasted (N = 6) *C*. *auratus*. Non-everted preparations were created by dividing the GIT into 8 equal length segments. Positive numbers indicate appearance in the serosal fluid suggesting mucosal-to-serosal movement. Data represent mean flux ± S.E.M. A two-way repeated measures ANOVA followed by a Holm-Sidak test was used (segment and fasting status as factors). No significant interaction effect was detected. Bars not sharing letters indicate significant differences (p<0.05).

The Mg^2+^ fluxes observed along the GIT sections for control diet animals (both fed and unfed) were one quarter to one half of the values observed when measured with Magnesium Ionophore VI compared to Magnesium Ionophore II (Fig 8). However, the previously observed increase with feeding was again present in all sections, with the esophagus demonstrating the largest increase followed by all other sections (Fig 8). As well, unfed animals displayed no zonation in flux (Fig 8).

**Fig 9:**
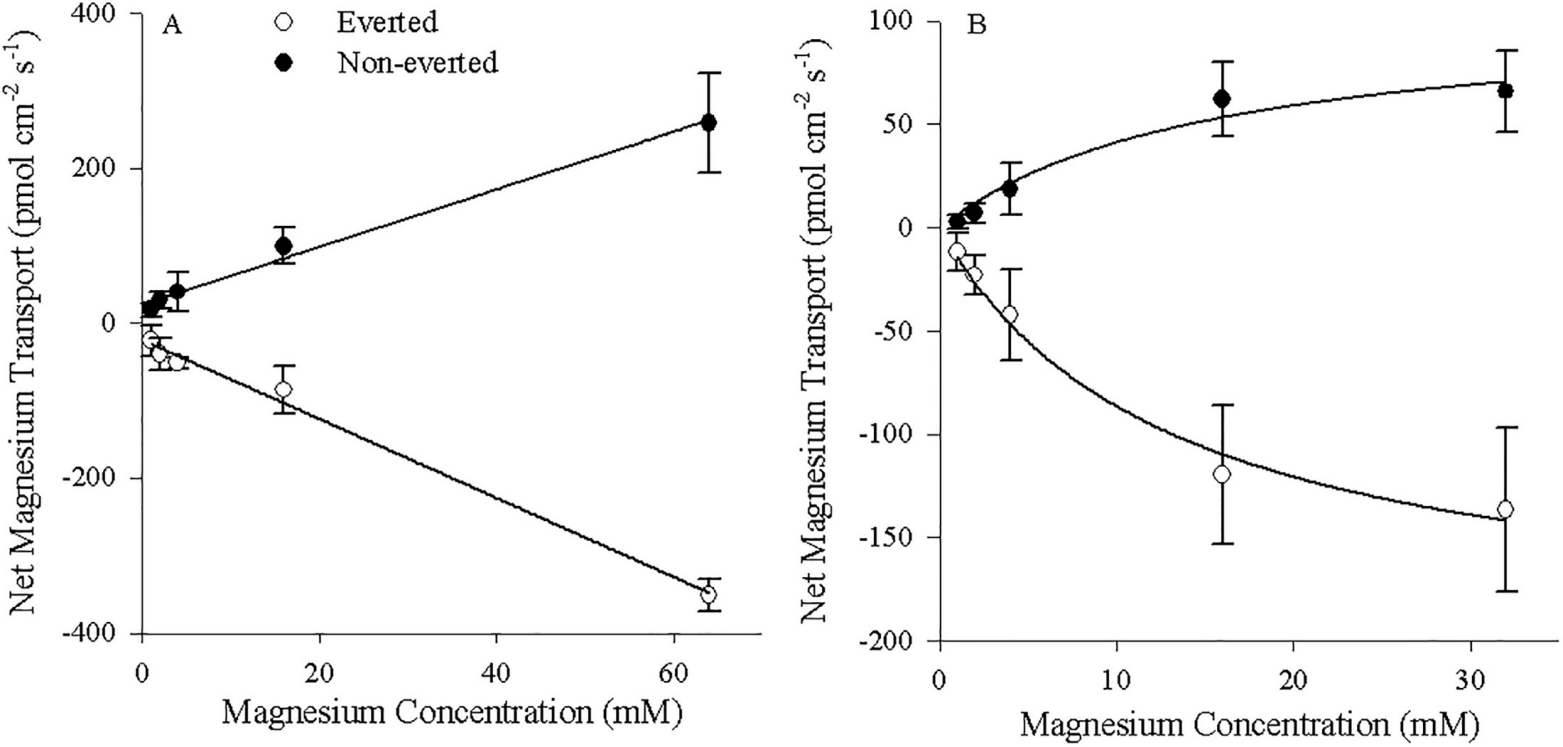
Transport kinetics by both everted and non-everted unfed tissues measured with magnesium ionophore VI. A) Esophageal and B) Rectal magnesium fluxes in the serosal fluid for non-everted (N = 4) and the mucosal fluid for everted (N = 4) tissue of unfed *C*. *auratus*. Cortland saline containing 0–64 mM MgSO_4_ was applied to the mucosal surface of the esophagus. Cortland saline containing 0–32 mM MgSO_4_ was applied to the mucosal surface of the rectum. Positive values in non-everted preparations indicate appearance in the serosal fluid (suggesting mucosal-to-serosal movement) and negative values in everted preparations indicate disappearance from the mucosal fluid (suggesting mucosal-to-serosal movement). Data represent mean flux ± S.E.M. Regression analysis revealed a significant (p<0.05) linear relationship between Mg^2+^ concentration and Mg^2+^ transport rate in the esophagus and a significant (p < 0.05) relationship between Mg^2+^ concentration and Mg^2+^ transport rate fitting a Michaelis-Menten curve with single saturation in the rectum. See text for more details.

Mg^2+^ transport into the serosal fluid by non-everted esophageal preparations (positive values) and out of the mucosal fluid by everted preparations (negative values) correlated linearly with alterations in the mucosal concentrations of Mg^2+^ (y = 3.71x + 24.1, (R^2^ = 0.991) and y = -5.09x − 22.0 (R^2^ = 0.993) respectively; p< 0.05; Fig 9A). The linear relationship was similar to that observed with magnesium ionophore II but the slopes and intercept values were smaller. Similarly, the relationship between Mg^2+^ transport and Mg^2+^ concentration within the non-everted rectal tissue preparations was again defined by the Michaelis–Menten equation f = 103.8x/(15.1 + x) (R^2^ = 0.961; Fig 9B) with Mg appearing in the serosal fluid. For the everted preparations, following conversion to positive values, the Michaelis–Menten equation f = 200.0x/(13.15 + x) similarly defined the correlation between Mg^2+^ transport and Mg^2+^ concentration (R^2^ = 0.988; Fig 9B). As with the esophagus the transport characteristics were maintained across ionophores, but the values (both J_Max_ and K_M_) decreased with magnesium ionophore VI.

## Discussion

### Application of SIET to GIT transport studies in teleost fish

This study presents evidence that the SIET is a useful tool for studying ion transport in the GIT of teleost fish, a novel approach that can complement existing approaches such as the Ussing Chamber and gut sacs. However, several caveats are applicable. As mentioned in the Materials and Methods, the Mg^2+^ ionophore II can also detect Ca^2+^ and H^+^ ions within the solutions. As such, changes in Mg^2+^ concentrations cannot be attributable to only Mg^2+^ unless all three ions are measured and show no interference with Mg^2+^ slope calculations as well as no change in concentration (Figs 3 and 4). Specifically, interfering ions may affect the slope when detecting Mg^2+^, and while this did not occur under the current experimental conditions, the Nicolsky–Eisenman equation provides a means for predicting the change in slope due to the change in voltage in the presence of an interfering ion. Application of this equation may be required under other experimental conditions where interference is detected. Additionally, if correlating changes are observed in Ca^2+^ and/or H^+^, no conclusions can be drawn as the source of the altered concentration could be attributable to two or more ions. During the measurements of Mg^2+^ transport kinetics, Mg^2+^ transport was altered and no corresponding changes in Ca^2+^ or H^+^ transport were observed (Figs 3 and 4). Thusly we can deduce characteristics of Mg^2+^ transport from the findings in the absence of interference discussed above. However, there were several instances where changes in Mg^2+^ flux were mirrored by changes in Ca^2+^; both Mg^2+^ and Ca^2+^ fluxes were simultaneously increased in the esophagus and anterior regions of the intestine (Fig 1), Mg^2+^ and Ca^2+^ transport in the rectum was altered from control when animals were fed a High Mg^2+^ diet (Fig 2), and ouabain reduced both Mg^2+^ and Ca^2+^ transport rates (Fig 5). In these instances, it is possible that only Ca^2+^ transport was changing and being detected by both ISMEs or Mg^2+^ and Ca^2+^ transport were both altered. However, deducing the contributions requires a different ionophore that is more selective for Mg^2+^ and/or not selective for Ca^2+^. This was demonstrated in Figs 8 and 9 using Mg^2+^ ionophore VI, which confirmed the trends observed in Mg^2+^ transport. It is important to note the magnitude of the ion fluxes measured with Mg^2+^ ionophore II (Figs 3 and 4) and Mg^2+^ ionophore VI (Figs 8 and 9). The decrease in magnitude could be attributed to the Mg^2+^ ionophore II detecting Ca^2+^ concentrations and overestimating Mg^2+^ concentrations. Overall, the data indicates that Magnesium ionophore VI is the superior choice for animal tissue studies for accuracy and simplicity.

### Zonation and impact of digestion with non-symmetrical and symmetrical conditions

Net Mg^2+^ and Ca^2+^ transport was uniform along the GIT of unfed animals however; zonation was apparent following the ingestion of a meal, with high rates in the anterior portions containing chyme (Figs 1A, 1B and 8). As diets contain high levels of divalent cations [40], this high rate of transport in the anterior segments may reflect increased diffusion down greater concentration gradients. However, mammalian intestines likewise show exaggerated Mg^2+^ transport rates in the proximal intestine at high luminal Mg^2+^ concentrations that were absent at low luminal Mg^2+^ concentrations [41]. Furthermore, increased anterior vs posterior Ca^2+^ transport was evident in tissues under symmetrical conditions (Fig 2B), indicating that while increased diffusion may contribute to transport rates while chyme is present, zonation remains in the absence of high luminal concentrations. Together this suggests that there may be two transport pathways operating at different luminal divalent ion concentrations; one that is active at low concentrations and another that is active at high concentrations. Additionally, while fluxes of Ca^2+^ and Mg^2+^ were variable along the intestine of rainbow trout, strong absorption was observed in the stomach [6]. *Carassius auratus* lack a stomach, and hence may require absorption along the anterior portion of the intestine to compensate. Ca^2+^ handling displayed further zonation, with elevated transport rates in the post-post section of fed animals (Fig 1B). This agrees with previous studies in the lake sturgeon where Ca^2+^ transport in the anterior intestine was primarily regulated by luminal concentrations, while distal segments were regulated by bodily requirement [42]. This suggests that fish may adjust transport in the posterior portion of the intestine to regulate dietary ion absorption to maintain homeostasis. However, as fed rates were not significantly different from unfed rates under symmetrical conditions (Fig 2A and 2B), this is likely not regulated during the digestion of individual meals, but over a longer time frame as seen with putative Mg^2+^ transporter mRNA expression [43].

Feeding resulted in a slight but non-significant increase in H^+^ flux rates over unfed rates in the anterior sections accompanied by a slight but non-significant decrease from unfed values in the mid sections. Combined, this resulted in a significantly higher rate of H^+^ flux into the anterior sections for fed animals, although not one that was significantly different from unfed animals (Fig 1C). Lacking a stomach, an anterior region associated with a large H^+^ excretion stimulated by digestion, as observed in the stomach possessing rainbow trout [44], was not expected. Furthermore, the FW-acclimated killifish, also lacking a stomach, displayed only marginal acidification of intestinal chyme during digestion as compared to fasting for 1–2 weeks, decreasing from pH 7.5 to pH 6.8 [45].

### Impact of dietary Mg^2+^ concentration

An increase in dietary Mg^2+^ decreased mucosal absorption and transitioned to mucosal secretion in the posterior tissues (Fig 2A). This was mirrored by a decrease in serosal secretion and transition to absorption (Fig 2A). The lack of influence on Ca^2+^ and H^+^ transport suggest that this alteration in transport can be attributed to changes in Mg^2+^ transport alone even though the ionophore detects both Ca^2+^ and H^+^. Despite Mg^2+^ transport and regulation being relatively poorly understood, there is an established body of literature underlining the importance of dietary Mg^2+^. Dietary Mg^2+^ (0.12–1.3 g kg^-1^ diet) is essential for the development and survival of freshwater fish [46], although the exact requirement is species- and environment-specific (e.g. rainbow trout 0.2 g kg^-1^ [46], channel catfish 1 g kg^-1^ [3]). Excess Mg^2+^ is also detrimental, as Mg^2+^ rich diets (above 745 mg kg^-1^) decreased growth and survival rate and activity of Na^+^-K^+^‐ATPase and Mg^2+^‐ATPase in juvenile gibel carp [47]. An increase in secretion in the distal intestine may be a physiological response to limit excess dietary Mg^2+^ absorption. The long-term regulation of a Mg^2+^ transporter, with a decrease in expression with low dietary Mg^2+^ and upregulation with high dietary Mg^2+^, was previously observed in the goldfish [43], suggesting that the secretion of Mg^2+^ is genetically controlled in response to dietary Mg^2+^. This may potentially occur only in the posterior portion of the intestine (Fig 2A).

### Kinetics and pharmacological effects on Mg^2+^ transport in the esophagus, rectum

In the esophagus a well-defined linear uptake from the mucosal fluid was present in the everted tissues, while a linear secretion into the serosal fluid was present in the non-everted tissues (Figs 3A and 9A), suggesting absorption and mucosal-to-serosal movement of Mg^2+^ when consuming the control diet. As well, the esophageal transport of ions was generally unaffected by the application of ouabain (Figs 5 and 6). In contrast, the low affinity absorption of Mg^2+^ in unfed rectal tissues showed Michaelis–Menten kinetics, with relatively high K_M_ values (13.5–19.9 mM) regardless of eversion (Figs 4A and 9B). Transport capacity (J_Max_) was, however, considerably higher, with an average rate of 212 pmol cm^−2^ s^−1^ in non-everted tissues compared to 520 pmol cm^−2^ s^−1^ in everted tissues (Fig 4A; or 103.8 pmol cm^−2^ s^−1^ to 200.0 pmol cm^−2^ s^−1^ using magnesium ionophore VI; Fig 9B). Furthermore, the rectal tissue was sensitive to ouabain application, which significantly reduced both Ca^2+^ and Mg^2+^ transport (Figs 5, 6 and 7). This response was exaggerated when applied to the mucosal fluid and surface, suggesting ouabain can more easily cross the mucosal epithelium of *C*. *auratus*. As both the everted and non-everted preparations showed no impact of Mg^2+^ concentration on Ca^2+^ or H^+^ transport (Figs 3B, 3C, 4B and 4C), the flux for both ions would interfere roughly equally at all Mg^2+^ concentrations. This indicates that the proposed kinetics of Mg^2+^ transport rates is reflective of Mg^2+^ transport alone, despite possible interference from other ions. However, the reduction in Mg^2+^ fluxes with ouabain application should be viewed with caution, as the ionophore may have been detecting the reduced fluxes of Ca^2+^ and overestimating the movement of Mg^2+^ (Figs 5, 6 and 7). Altogether, the evidence collected suggests the presence of two Mg^2+^ transport pathways: one linear diffusive paracellular pathway that is conceivably passive in the esophagus, and one saturable transcelluar pathway, that is conceivably active in the rectum. Previous studies have supported this two-pathway model in both mammals [48, 49] and fish [8], but the zonation of the two is novel.

Passive Mg^2+^ transport rates likely depend on intestinal tight junction composition and claudin expression [50]. Interestingly, C. auratus displays spatial zonation in claudin expression along the GIT; with lowest expression in the anterior intestine and highest expression in the posterior intestine [51]. This expression pattern correlates well with the observed high rate of passive transport in the anterior portions of the goldfish intestine (Figs 1A, 4A, 9A) which are reduced in the posterior portions (Figs 1A, 5A and 9B). In contrast, the posterior portions indicate a reliance on transcellular transport, likely accomplished by Mg^2+^ entry down its electrochemical gradient across the apical enterocyte membrane through a channel (suspected to be formed by transient receptor potential melastatin (TRPM) 6 and 7; [52, 53]). It is then extruded across the basolateral membrane through a secondary active, Na^+^-dependent and ouabain-sensitive transcellular absorptive mechanism (e.g. [2, 20]). This process has long been hypothesized to occur via a hypothetical Na^+^/Mg^2+^ exchanger (NME) localized to the basolateral membrane [20, 21]. Recently, the solute carrier 41a1 (SLC41a1) has been suggested as the putative NME in mammals [33, 54, 55] and fish [43, 56]. Interestingly, SLC41a1 transcript levels were highest at the rectum in goldfish, similar to mammals [33, 43] and TRPM6 zonation [52]. A possible explanation for this trend is that as passive transport decreases along the GIT with decreased concentration gradients there is a greater reliance on active transport, and therefore active transporters such as SLC41a1 are more highly expressed [41, 57]. Alternatively, if SLC41a1 secretes Mg^2+^ across the apical membrane [43], it could reduce the measured Mg^2+^ flux, possibly explaining the lowest transport at the rectum (Figs 1A and 8) and the reversal in flux direction at high dietary Mg^2+^ levels (Fig 2A). The ouabain-induced reduction in fluxes (Figs 5, 6 and 7) is likely due to inhibition of the basolateral Na^+^-K^+^-ATPase, which required to maintain the high Na^+^ gradient driving Mg^2+^ transport via the NME [2, 8].

Surprisingly, Co3Hex not only failed to exhibit synergistic effects with ouabain, but also failed to exhibit any response on its own in terms of Mg^2+^ fluxes (Figs 5, 6 and 7). In contrast, Co3Hex reduced inward Mg^2+^ fluxes by over 50% in HEK-293 cells, although the HEK-293 cells were incubated in a higher Mg^2+^ saline (10mM or ~5 times higher than the mucosal concentration in the present experiment), and exposed to a ~10 fold higher concentration of Co3Hex (1mM; [33]). Furthermore, Co3Hex inhibited magnesium transport in apical BBMVs isolated from the proximal intestine of *Oreochromis mossambicus*, where Mg^2+^ transport obeyed Michaelis–Menten kinetics and was strongly temperature dependent, indicative of a carrier mechanism [21]. It is possible that the observed paracellular transport in the intact esophageal tissue of *C auratus* obscured additional carrier mediated transport, however the lack of effect in the rectum suggests that the Mg^2+^ being transported may not be hydrated [34, 35] as Co3Hex mimics the first hydration shell of Mg^2+^.

## Conclusions

We created the opportunity to study ion transport in model organisms and life stages that were previously unavailable by combining the benefits of gut sacs (simultaneous studies of the GIT without disturbing the mucous layer of the tissue, fast preparation times, and minimal training), with the benefits of SIET (fine-scale resolution of transport, ability to study small animals and tissues, ionophores for ions lacking feasible radioisotopes). Specifically, using this approach we provided the first indication of a zoned, functional role for the GIT in Mg^2+^ and Ca^2+^ transport and not H^+^ in *C*. *auratus*, supporting previous *in vivo* work in other fish species (e.g. [6, 7, 8, 9, 10, 45]). Furthermore, this study used SIET to provide measurable impacts of digestion and diet, suggesting excess dietary Mg^2+^ was secreted across the posterior portion of the GIT but that transport rates during the digestion of a control diet were not altered. Finally, mechanistic characterization of Mg^2+^ transport along the GIT provided the evidence of zoned Mg^2+^ transport kinetics in the intestine of *C*. *auratus*. The transport kinetics of Mg^2+^ in the esophagus were similar to passive, paracellular transport, while evidence suggested an energy-dependent, saturable mechanism of Mg^2+^ uptake in the rectum. These are the first quantifications of ion transport along the GIT of *C*. *auratus*, and offer evidence for a novel method for teleost ion transport studies. The results suggest that careful consideration must be made when choosing ionophores, in particular Magnesium Ionophore VI is suggested for use when studying tissues and/or cells bathed in solutions containing interfering ions such as physiological salines.
